# Cytometric and genetic analysis of Slovenian local apple genotypes

**DOI:** 10.1186/s12870-025-07382-0

**Published:** 2025-10-08

**Authors:** Kristina Gostinčar, Gregor Osterc, Nataša Štajner, Zlata Luthar

**Affiliations:** Department of Agronomy, Biotechnical faculty, University of Ljubljana, Ljubljana, 1000 Slovenia

**Keywords:** *Malus* x *domestica *Borkh., Apple, Genetic resources, Genetic diversity, SSR, Flow cytometry, Ploidy

## Abstract

**Background:**

The genetic diversity and ploidy of apple varieties in Slovenia is not well known so far. In this study, a total of 233 genotypes and 8 reference varieties were analysed with a set of 15 SSR markers in order to estimate the genetic diversity and to determine the genetic structure and relationships among the apple germplasm studied.

**Results:**

A total of 228 different alleles were successfully amplified, with an average of 15.2 alleles per locus. All markers were highly discriminating and powerful for varietal identification, with an average power of discrimination of 0.94. The results showed that the germplasm is highly diverse with a mean expected heterozygosity of 0.83 and a mean observed heterozygosity of 0.86. Of the 233 genotypes, 51 were recognised as synonyms and duplicates (22% redundancy), and a total of 182 unique genotypes were identified in the collections. An amount of 23% of the genotypes were identified as triploids by flow cytometry. The UPGMA clustering revealed three main groups. The genetic analysis, which was performed using the Bayesian model, revealed a very strong differentiation into two main groups. The first group comprised 85 genotypes (72% with *qI* > 0.80), including four reference varieties and seven local varieties. The second group comprised 105 genotypes (65% with *qI* > 0.80), including 4 reference varieties and two local varieties. In addition, nested Bayesian clustering was applied to these two groups, and four and two sub-groups were found, respectively.

**Conclusions:**

Altogether, this study of apple germplasm in the Slovenian landscape indicate a high diversity of apple varieties present and underline the importance of preserving these varieties. The SSR markers used, helped to identify duplicates, synonyms and homonyms within and between collections, which will help us to establish a core collection.

## Background

One of the most economically important fruit crops grown in the temperate regions of the world is the domesticated apple (*Malus x domestica* Borkh.) [1], which is also the most important fruit variety in Slovenia [2]. In 2023, more than 97 million tonnes of apples were produced around the world and more than 17 million tonnes in Europe [3]. In 2024, more than 50 tonnes were produced in Slovenia [2]. In addition to their economic importance, apples play an important role in culture, art, folklore and nutrition due to their nutritional value [4]. Most apple varieties are diploid (2n = 2x = 34), but there are also some triploid (2n = 3x = 51) and tetraploid varieties (2n = 4x = 68; [5]. Although *M. seiversii* M. is considered the main contributor to the *Malus* x *domestica* gene pool, domestication is the result of a long process of hybridization events between different wild apple species, including *M. baccata* (L.) Borkh., *M. orientalis* Uglitz. and *M. silvestris* (L.) Miller [1, 6], which has resulted in 10,000 apple cultivars worldwide [7]. However, apple breeding programmes are based on a few well-adopted genotypes, e.g. Jonathan, Cox’s Orange Pippin, Red Delicious, Golden Delicious and McIntosh [8, 9]. In addition, apple production since the second half of the 20th century, has been based on the reduced number of varieties, such as Idared, Gala, Elstar, Jonagold and Cripps Pink [10]. The main apple varieties currently grown in commercial orchards are Golden Delicious, Gala, Idared, Red Delicious, Jonagold, Granny Smith, Elstar [11]. As a result, traditional and locally well-adopted varieties have been replaced by limited and related varieties, which combined with vegetative practices based on cuttings and grafting, reduces genetic diversity in orchards and may hinder future apple breeding [12].

In recent years, it has been recognised that old apple varieties can be used as resources to respond to pests, pathogens and climate change, which has led to various measures to preserve apple genetic resources around the world [13]. Many apple collections preserve old varieties that were traditionally grown in their regions, as well as varieties from different geographic origins that were introduced a long time ago. Originally, the formation of germplasm collections focussed mainly on morphology, eco-geography and passport information [14]. However, the termination of varieties based on phenotypic traits is difficult, as these depend on environmental factors [7]. Genotyping of gene bank collections is necessary to verify the identity of accessions, detect mislabelled accessions, genetic diversity and pedigrees. As germplasm collections are expensive to maintain, genotyping of genetic resources is crucial for gene banks to create core collections that preserve maximum genetic diversity while minimizing the repetition of apples [15].

Molecular markers play a crucial role in the identification, preservation and utilization of plant genetic resources [16, 17]. Based on microsatellite markers (SSR), the diversity of apple collections has been assessed in numerous research [18–21]. Microsatellites have proven to be the markers of choice due to their high level of polymorphism, codominance, abundance in the genome, reproducibility and low cost. In addition, molecular markers can be a useful tool to localise duplicates as well as potential synonymies and/or homonymies [22]. For the study of apple resources, the *Malus/Pyrus* Working Group of the European Collaborative Programme for Crop Genetic Resources (ECPGR) has recommended a set of markers to harmonize data between different groups. The set has changed over time as more knowledge about polymorphisms became available. Nowadays, the ECPGR set includes 17 SSRs that cover the most of the apple genome and have been tested on a set of standard apple accessions [23, 24].

In Slovenia, work on apple genetic resources has so far been limited to their preservation in the national repository of the Slovenian Plant Gene Bank and to phenotypic descriptions of varieties. However, molecular identification has not yet been conducted. The present study is the first to determine the genetic diversity and identity of *Malus* accessions from three different locations in Slovenia, as well as investigate the presence of duplicates, synonyms and homonyms in order to improve the management of the collections.

## Materials and methods

### Plant material

Apple germplasm collections from three different locations in Slovenia were included in this study. Of these 233 genotypes, 118 genotypes were collected in Pleterje (45°49’04.3"N, 15°21’17.6"E), which is part of the national repository of the Slovenian Plant Gene Bank, 113 genotypes were collected in Kozjansko (46°02’38.6"N, 15°35’26.6"E), a regional park in eastern Slovenia and 2 genotypes were collected in Kojsko (46°00’18.1"N, 13°34’31.5"E) as part of a private collection. This study also included eight reference varieties (*Malus domestica* Red Delicious, Fiesta, Worcester Pearmain, Prima, Michelin, Malling 9, *Malus floribunda 821*, *Malus robusta*), as recommended by the European Collaborative Programme for Crop Genetic Resources (ECPGR) *Malus/Pyrus* Working Group [25], to allow internal data harmonization and further comparison of results with other studies. The DNA samples of the reference varieties were obtained from INRA, Angers, France.

### DNA extraction and SSR fingerprinting

Total genomic DNA was extracted from young leaf tissue using a cetyltrimethylammonium bromide (CTAB) protocol according to Kump et al. [26], with the modification of treating the supernatant with chlorophorm-isoamyl alcohol (24:1) instead of phenol-chlorophorm isoamyl alcohol (25:24:1). The DNA samples were quantified using the DyNA Quant 200 fluorometer (Amersham Biosciences, Chicago, ZDA), and the quality was checked using a spectrophotometer (NanoVue Plus, GE Healthcare) based on the absorbance ratio at the wavelengths A260/A280 and A260/A230.

Apple accessions were genotyped with a set of 15 SSR markers developed by different groups [27–30] (Table 1). In addition, 14 SSR markers are recommended by the ECPGR *Malus/Pyrus* working group [23]. PCR amplification was performed as described by Urresterazu et al. [24] and SSR amplification products were analysed using an ABI 3130XL (Applied Biosystems, Carlsbad, CA, ZDA). Allele sizes were determined using GeneMapper 5 (Applied Biosystems, Foster City, CA, USA).

**Table 1:** SSR used for the characterisation of apple accessions. Locus, multiplex (MP), fluorochrome, size range of the amplified fragment, primer sequence, concentration

Locus	MP	Dye	Size range (bp)	Forward primer sequence 5′→3′	Reverse primer sequence 5′→3′	Primer concentration
Hi02c07 ^a^	MP_3_	VIC	104–154	agagctacggggatccaaat	gtttaagcatcccgattgaaagg	[0.09 µM]
CH-Vf1 ^d^	MP_2_	VIC	133–188	atcaccaccagcagcaaag	gtttcttcatacaaatcaaagcacaaccc	[0.09 µM]
CH02c06 ^b^	MP_1_	NED	201–286	tgacgaaatccactactaatgca	gtttgattgcgcgctttttaacat	[0.36 µM]
CH05f06 ^b^	MP_2_	PET	165–197	ttagatccggtcactctccact	gttttggaggaagacgaagaagaaag	[0.18 µM]
CH03d07 ^b^	MP_4_	6-FAM	169–232	caaatcaatgcaaaactgtca	gtttggcttctggccatgatttta	[0.27 µM]
CH04e05 ^b^	MP_2_	6-FAM	178–232	aggctaacagaaatgtggtttg	gtttatggctcctattgccatcat	[0.18 µM]
CH01h10 ^b^	MP_1_	VIC	90–144	tgcaaagataggtagatatatgcca	gtttaggagggattgtttgtgcac	[0.18 µM]
CH01f03b ^b^	MP_1_	6-FAM	141–200	gagaagcaaatgcaaaaccc	gtttctccccggctcctattctac	[0.18 µM]
CH02c11 ^b^	MP_3_	NED	207–269	tgaaggcaatcactctgtgc	gtttttccgagaatcctcttcgac	[0.27 µM]
CH02d08 ^b^	MP_2_	NED	209–262	tccaaaatggcgtacctctc	gtttgcagacactcactcactatctctc	[0.18 µM]
CH01f02 ^b^	MP_3_	6-FAM	163–227	accacattagagcagttgagg	gtttctggtttgttttcctccagc	[0.18 µM]
GD147 ^c^	MP_3_	PET	122–166	tcccgccatttctctgc	gtttaaaccgctgctgctgaac	[0.18 µM]
CH04c07 ^b^	MP_4_	VIC	98–148	ggccttccatgtctcagaag	gtttcctcatgccctccactaaca	[0.18 µM]
CH02c09 ^b^	MP_4_	NED	227–261	ttatgtaccaactttgctaacctc	gtttagaagcagcagaggaggatg	[0.18 µM]
CH01h01 ^b^	MP_1_	PET	104–150	gaaagacttgcagtgggagc	gtttggagtgggtttgagaaggtt	[0.18 µM]

^a^[25﻿], ^b^[24], ^c^[23], ^d^[26]

### Flow cytometry

Ploidy was determined for each genotype with a flow cytometer using a known diploid ‘Topaz’ as an internal standard. Apart from some minor adjustments, samples were prepared according to the manufacturer’s instructions. In brief, 500 µL of CyStain UV OxProtect buffer with DAPI (Sysmex, Germany) was added to a Petri dish containing a small piece of the leaf sample and the internal standard and then were chopped. The mixture was then filtered through a 30 μm CellTrics filter (Sysmex, Germany) into a sample tube. Flow cytometry was performed using a CyPloidy Analyzer (Sysmex, Germany). Flow cytometry was performed for 182 genotypes. The reference varieties were not analysed by flow cytometry.

### Data analysis

To establish the uniqueness of each accession, the multilocus SSR profiles were compared pairwise using the CERVUS version 3.07 software package [31]. Accessions were considered duplicates if they had identical SSR profiles with an allelic difference for at most two SSR loci [18, 24]. Redundant accessions were excluded from further genetic analysis to avoid bias in the genetic analyses. Analyses of the descriptive diversity statistics were performed for each locus using the software SPAGeDi v.1.3 [32] for diploid and triploid genotypes together. For each locus, the software calculated the number of alleles per locus (NA), the number of effective alleles (Ne), the percentage of rare alleles (frequency < 0.01), the observed (H_o_), the expected (H_e_) heterozygosity and the individual inbreeding coefficient (Fi). Discrimination power (PD) was calculated according to Tessier et al. [33] and polymorphic information content (PIC) according to Botstein et al. [34].

The genetic structure of the unique genotypes was assessed using a clustering algorithm based on a Bayesian model implemented in STRUCTURE v.2.3.4 software [35]. We used the recessive allele approach to analyse diploids and triploids together. The analysis was performed according to the admixture model with correlated frequencies. The simulation was performed with 10 runs for each proposed K value [1–10], and each of these runs was implemented with ‘200,000’ burn-in interactions, followed by another ‘500,000’ Markov chain Monte Carlo iterations [18, 24, 36]. The results obtained were analysed with Structure Selector [37]. To determine the best K value supported by the data, we used the ΔK method described by Evanno et al. [38]. If the results indicated that the K groups could be further structured into sub-groups, a second level (nested) structural analysis was performed individually for each K group [39, 40]. Genotypes were assigned to the group or sub-group whose membership coefficient (*qI*) was higher than 0.8 [21, 41–43]. Similarity between unique genotypes was also performed using an unweighted pair group method with Darwin software v 6.0.21. Darwin software was also used to perform principal coordinate analysis (PCoA) [44].

## Results

### SSR polymorphism and genetic diversity

In this study, a total number of 233 apple genotypes were genotyped using a set of 15 SSR markers. Based on the molecular data, 51 synonymous genotypes (22%) were detected, which were therefore not included in the subsequent analysis of genetic diversity and structure to avoid bias in genetic analyses. Among the 51 synonyms, 20 groups of duplicates (accessions with the same name and genotype, but grown in different locations) and 21 groups of synonyms (accessions with different names and the same genotype) were identified.

All SSR markers were polymorphic. The primer pair CH02c11 amplified two loci but the secondary locus was monomorphic, as reported by other authors [45, 46], and only the amplification of the main locus was considered. A total of 228 alleles were amplified with an average of 15.2 per locus (Table 2), ranging from 10 (CH02c09) to 23 (CH01f02), but the number of effective alleles per locus was lower (Ne = 6.47). With the exception of locus CH09c09, the other 14 loci contained rare alleles (N_R_; frequency less than 1%), ranging from 2 (GD147) to 9 (CH01f02) and becoming more frequent as the number of alleles per locus increased. The 15 loci had a polymorphism information content (PIC) ranging from 0.64 (CH-Vf1) to 0.89 (CH02c11), with a mean of 0.80. The power of discrimination (PD) has an overall mean of 0.94 (ranging from 0.86 to 0.98), indicating that the loci are sufficiently polymorphic in discriminating individuals. With an average value of 0.83, the expected heterozygosity (He) of the SSR ranged from 0.70 (CH01h10, CH-Vf1) to 0.90 (CH02c11). The observed heterozygosity (Ho) varied from 0.73 (CH-Vf1) to 0.94 (CH02c11), with a mean value of 0.86.

**Table 2:** Measures of genetic diversity of 182 unique genotypes. Locus, number of alleles (NA), number of effective alleles (Ne), number of rare alleles (N_R_), expected (He) and observed (Ho) heterizigosity, power of discrimination (P_D_), polymorphsm information content (PIC) and inbreeding coefficient (Fi)

Locus	NA	Ne	*N*_*R*_	He	Ho	PD	PIC	Fi
CH02c06	20	9.04	5	0.89	0.83	0.98	0.88	0.047
CH01h10	12	3.3	5	0.70	0.83	0.86	0.65	−0.195
CH01f03b	13	4.57	6	0.78	0.87	0.92	0.75	−0.136
CH01h01	14	7.13	4	0.86	0.88	0.96	0.84	−0.042
CH-Vf1	14	3.29	4	0.70	0.73	0.86	0.64	−0.065
CH05f06	11	5.62	7	0.82	0.84	0.95	0.80	−0.041
CH04e05	17	4.18	8	0.76	0.79	0.91	0.73	−0.047
CH02d08	18	6.58	6	0.85	0.90	0.96	0.83	−0.072
Hi02c07	12	5.36	4	0.81	0.83	0.94	0.79	−0.04
CH02c11	17	10.37	5	0.90	0.94	0.98	0.89	−0.052
CH01f02	23	9.3	9	0.89	0.88	0.98	0.88	−0.002
GD147	13	6.25	2	0.84	0.83	0.96	0.82	−0.021
CH03d07	19	7.77	8	0.87	0.89	0.97	0.86	−0.034
CH04c07	15	7.61	4	0.87	0.92	0.97	0.85	−0.063
CH02c09	10	6.73	0	0.85	0.92	0.96	0.83	−0.099
Mean	15.2	6.47	5.1	0.83	0.86	0.94	0.80	−0.055

### Accession identification

Pairwise comparison of the SSR profiles revealed 37 groups of genotypes that had the same SSR profile (marker duplicates). The size of these groups, totalling 88 genotypes, ranged from two to six accessions. As a result, 20 groups of duplicates were identified (Weisser Klarapfel, Steirischer Maschanzker, Gravensteiner, Charlamowsky, Geflammter Cardinal, Mollies Delicious, Harberts Renette, Easygro, Goldparmane, Jonathan, Kronprinz Rudolph, Jakob Lebel, Kanada Renette, Gloria Mundi, Bleinheim Renette, Bohnapfel, Glockenapfel, Ontario, Krumstiel, Petnošter). In addition, there were 21 groups in which the names of the genotypes were different or their names were unknown, as shown in Table 3. In two groups the synonyms originated from both locations, in 8 groups the synonyms originated from a different location and in 13 groups the synonyms originated from the same location.

**Table 3:** Groups of synonyms

Group	Synonyms- same genotype and different name			
1	Weisser Klarapfel	Weisser Winterkalvil	Štajerski Pogačar	Steirscher Maschanzker
2	Geflammter Cardinal	Zgodnja Kavčič	Repuvšca	Rambura
3	Rote Sternrenette	Bobovača	Bogatinka	
4	Nova Mac	Obilnaja		
5	Roter Astrachan	Jakobšca		
6	Zgodnja Kavčič	Muškatevka		
7	Harberts Renette	Grosse Casseler Renette		
8	Vivanka	Žlahtnica		
9	Blaucher Wadenswil	Dolenjska Voščenka		
10	Kronprinz Rudolph	Steirischer Maschanzker		
11	Bellefleur	Rumena Lepocvetka		
12	Zeleno zimsko jabolko	Debela Vahna		
13	Zgodnje zimsko Kavčič	London Pepping		
14	Ontario	Pohorka		
15	Solnečna	Sevastopolska		
16	Koniginapfel	Orleanska Reneta		
17	Paplerjev Bobovec	Jesenska Rožmarinka		
18	Lovrenčovka	Rožmarinka		
19	Bartolenka	Detel		
20	Jesenska Tafelca	Glassapfel		
21	Roter Astrachan	Julyred		

In addition, 26 groups of homonyms (same name, different genotype) were found in this study: Roter Astrachan, Weisser Klarapfel, Harberts Renette, Mcintosh, Blauacher Wadenswil, Ananasrenette, Spartan, Bismarckapfel, Obilnaja, Solnečna, James Grieve, Burgundy, Baumanns Renette, Weisser Winterkalvil, Cox’s Orange Renette, Ananasrenette, Dolenjska Voščenka, Reinette de Boskoop, Štajerski Pogačar, Obilnaja, London Pepping, Rožmarinka, NY 623,452, Pesnica, Steirscher Maschanzker and Zgodnja Kavčič. The number of trees with different homonyms varies between 2 and 4, with 20 groups with two genotypes, 5 groups with three genotypes and one group of homonyms with 4 genotypes. Furthermore, in 15 groups of homonyms the genotypes originate from the same location and in 11 groups from different locations (Table 4).

**Table 4:** Homonyms present in the same and different locations

Location	Homonyms
Same	Harberts Renette, Mcintosh, NY 623,452, Steirscher Maschanzker, Blauacher Wadenswil, Ananasrenette, Spartan, Bismarckapfel, Obilnaja, Solnečna, Burgundy, Zgodnja Kavčič, Pesnica, Obilnaja, Rožmarinka
Different	Roter Astrachan, Weisser Klarapfel, James Grieve, Baumanns Renette, Weisser Winterkalvil, Cox’s Orange Renette, Ananasrenette, Dolenjska Voščenka, Reinette de Boskoop, Štajerski Pogačar, London Pepping

### Genetic structure analysis

**Fig. 1:**
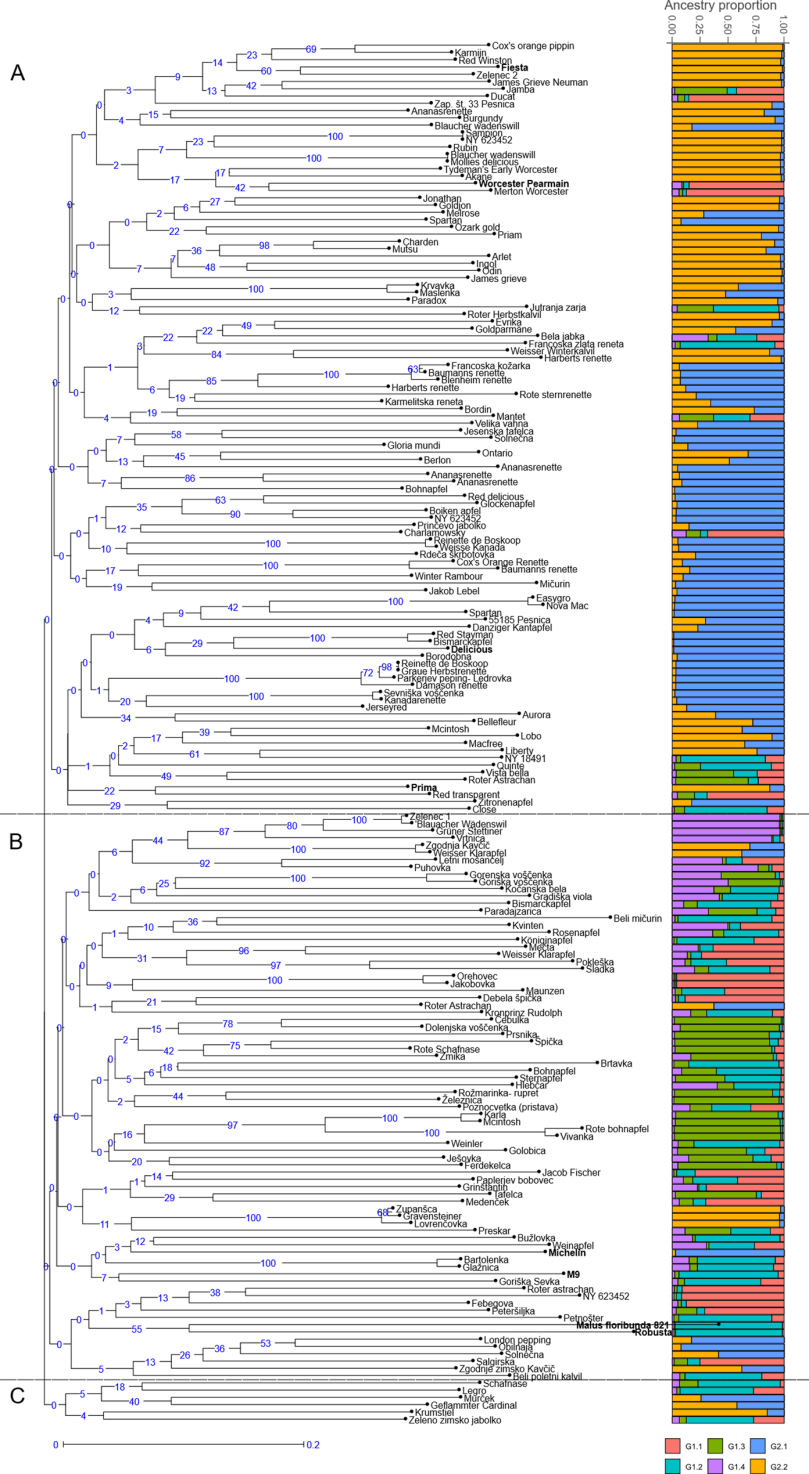
**A** UPGMA dendrogram showing the genetic relationships between 182 genotypes and 8 reference varieties (in bold). **B** Group probabilities obtained for *K* = 2 by Bayesian clustering and its sub-groups. Each bar represents the genetic background of an individual according to the proportion derived from each of the six sub-groups

The genetic relationship between the analysed genotypes is represented by UPGMA clusters (Fig. 1). Neighbour-joining tree analysis identified three main groups comprising both diploid and triploid unique genotypes. Group A comprises 106 genotypes, including four reference varieties (Fiesta, Worcester Pearmain, Delicious, Prima), 78 genotypes from Pleterje and 24 from Kozjansko. This group also includes two local varieties, Zelenec 2 and Velika Vahna. It also includes five traditional varieties, Jonathan, Melrose, Ontario, Bohnapfel and Red Delicious. Group B includes 78 genotypes, 23 genotypes from Pleterje and 51 from Kozjansko as well as four reference varieties (Michelin, M9, *Malus floribunda 821*, Robusta). This group includes seven local varieties Zelenec 1, Gorenska Voščenka, Vivanka, Karla, Dolenjska Voščenka, Goriška Sevka and Paplerjev Bobovec as well as a traditional variety Kronprinz. Group C contains 6 genotypes, 3 genotypes from Pleterje, 3 from Kozjansko and none reference variety. It includes a traditional variety Krumstiel.

In addition, a Bayesian analysis of the genetic structure of 182 unique genotypes and 8 reference varieties was performed with STRUCTURE 2.3.4 using K values between 1 and 10, with ten independent assessments of the log likelihood of the data for each *K* value. The results of the analysis were processed with Structure Selector and showed the most probable hierarchy division into two groups (K = 2, ΔK = 35.5) (Fig. 1). This partition reflected that the analysed material could be divided into two main groups, one comprising 81 genotypes (G1) and the other 111 genotypes (G2). In addition, we decided to perform a nested-Bayesian clustering in which each genotype from group K = 2 was analysed individually. Analysing the relationships between K and ΔK for G1 and G2 resulted in maximum ΔK values at K = 4 for G1 and K = 2 for G2. The descriptive statistics for these final sub-groups obtained in this study are shown in Table 5. The classification of genotypes with *qI* > 0.80 was 72% for G1, 65% for G2, 43% for G1.1, 21% for G1.2, 55% for G1.3, 50% for G1.4, 78% for G2.1, 72% for G2.2. Group G1.1 contained 21 genotypes and one reference variety (Worcester Pearmain), most of which belong to cluster B in the UPGMA dendrogram. This group contains a local variety Paplerjev Bobovec and other international varieties such as Merton Worcester, Jakob Fischer and Roter Astrachan. Admix varieties in this group are Mečta, Charlamowsky, Grinštantin, Pokleška and Maunzen. Group G1.2 contains 34 genotypes and three reference varieties (M9, Robusta, *M. floribunda 821*), most of which belong to cluster B in the UPGMA dendrogram. This group includes the local variety Goriška Sevka and international varieties such as Close, Rožnik, Kronprintz Rudolph and Bismarckapfel. Admix varieties in this group are Gradiška Viola, Jutranja Zarja, Poznocvetka, Bela jabka, Sladka and Bohnapfel. Group G1.3 contained 22 genotypes and no reference variety, most of which belong to cluster B in the UPGMA dendrogram. This group includes the local varieties Karla, Vivanka, Gorenska Voščenka and Dolenjska Voščenka as well as international varieties like Roter Astrachan, Rote Schafnase, McIntosh and Rote Bohnapfel. Admix varieties in this group are Preskar, Ješovka, Paradajzarica, Jamba and Vista Bella. Group G1.4 contained 8 genotypes and no reference variety, most of which belong to cluster B in the UPGMA dendrogram. A local variety Zelenec 1 and international varieties such as Blauacher Wadenswil, Gruner Stettiner and Vrtnica are represented in this group. Admix varieties in this group are Kvinten, Letni Mošancelj and Goriška Voščenka. Group G2.1 contains 55 genotypes and two reference varieties (Michelin and Delicious), most of which belong to cluster A in the UPGMA dendrogram. This group includes the local variety Velika Vahna, some traditional varieties such as Melrose, Bohnapfel and Red Delicious and some international varieties such as Ananasrenette, Baumanns Renette, Bleinheim Renette, Harberts Renette, Kanadarenette, Damason Renette, Renette de Boskoop and Glockenapfel. Admix varieties are Maslenka, Aurora, Solnečna and Karmeliter Renette. Group G2.2 contains 50 genotypes and two reference varieties (Prima and Fiesta), most of which belong to cluster A in the UPGMA dendrogram. This group includes the local variety Zelenec 2, some traditional varieties such as Jonathan, Ontario and Krumstiel. Some of the international varieties that belong to this group are: Gravensteiner, Cox’s Orange Pippin, Karmijn, Red Winston, Sampion, Mollies Delicious, James Grieve and Rother Herbst Calvil. Admix are Goldparmane, McIntosh, Zgodnja Kavčič, Geflammter Cardinal and Weisser Klarapfel.

**Table 5:** Genetic diversity measures for each of the genetic groups and sub-groups defined with STRUCTURE. Number of varieties (n), number of reference varieties (nR), percentage of genotypes with qI > 0.80 to the group, number of local and traditional varieties, number of alleles (N_A_), number of alleles per locus (N_A_/locus), expected heterozygosity (He)

Genetic Group	*n*	nR	% qI > 0.80	*n* of local varieties	*N*_A_	*N*_A_/locus	He
G1	85	4	72	7	237	15.8	0.85
G2	105	4	65	8	180	12.0	0.78
G1.1	21	1	43	1	122	8.1	0.80
G1.2	34	3	21	1	220	14.7	0.88
G1.3	22	0	55	4	120	8.0	0.76
G1.4	8	0	50	1	69	4.6	0.69
G2.1	55	2	78	4	153	10.2	0.80
G2.2	50	2	72	4	139	9.3	0.78

**Fig. 2:**
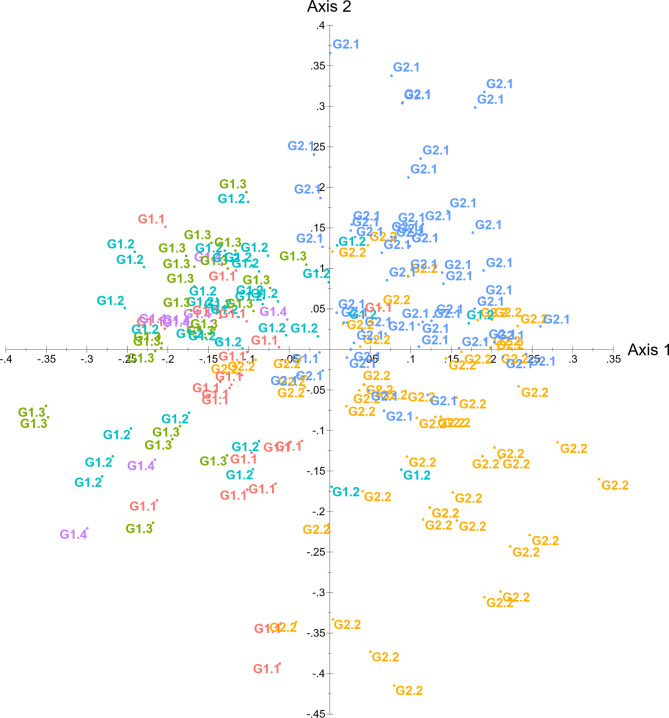
Principal coordinate analysis (PCoA) based on 15 SSR loci of 182 genotypes and 8 reference varieties using DARwin software. The colour codes are based on grouping in Bayesian clustering

Finally, a multivariate principal coordinate analysis (PCoA) performed in DARwin confirmed the genetic discrimination between the groups. The six sub-groups revealed by the structural analysis were distinguished using the PCoA plot, with the two-principal axes (one and two) explaining 5.04% and 4.59% of the variation (Fig. 2).

### Ploidy

SSR peak analysis revealed the presence of polyploidy within the apple germplasm. Fifty-two of the 182 genotypes analysed (28%) had at least three triallelic loci, indicating a triploid nature. Among the analysed loci, CH01h10 had three alleles in all loci of at least 10 genotypes. In addition, locus CH02c11 had three alleles in 46 genotypes, demonstrating its high sensitivity for the detection of triploid genotypes. The distribution of triallelic loci varied between genotypes, with some genotypes showing triallelic patterns across multiple loci, such as Rožnik with three alleles at 10 loci, while others show this pattern at fewer loci, such as Zupanšca with three alleles at 4 loci.

To confirm the presence of triploid genotypes, flow cytometry was performed. Among 182 genotypes analysed, 41 triploids were identified, corresponding to 23% of all genotypes analysed (Table 6). All genotypes predicted as triploids by SSR analysis were confirmed by flow cytometry.

**Table 6:** Triploid genotypes identified by flow cytometry

Triploid genotypes				
Close	Jakob Lebel	Velika Vahna	Pesnica	Štajerski Pogačar
Gravenstainer	Kanadarenette	Graue Herbstrenette	Sevniška Voščenka	Lovrenčovka
Geflammter Cardinal	Gruner Stettiner	Bismarckapfel	Boiken Apfel	Legro
Zgodnja Kavčič	Karmeliter Renette	Bordin	Koniginapfel	Glažnica
NY 623,452	Blenheim Renette	Reinette de Boskoop	Damason Renette	Baumanns Renette
Harberts Renette	Zeleno Zimsko Jabolko	Red Stayman	Reinette de Boskoop	Hlebčar
Mutsu	Bohnapfel	Charden	Jakob Fischer	Graue Herbstrenette
Zupanšca	Rdeča Škrbotovka	Jakobovka	Grinštanin	Rožnik
Blauacher Wadenswil	Dolenjska Voščenka	Weisse Kanada	Roter Stettiner	Schafnase

## Discussion

The accurate identification of genotypes in gene banks by molecular markers, such as microsatellites, has improved the management of collections by identifying duplicates, synonyms and homonyms, as well as understanding the origin of varieties, and establishing the importance of introgression, polyploidy and hybridization in their development [19, 45, 47–49]. Genotypes of apples conserved in the national programme of the Slovenian Plant Gene Bank in the national repository Pleterje were included in this study. In order to include as many different genotypes found in the field as possible, old local varieties maintained in the Kozjansko regional park and varieties from a smaller private collection Kojsko were also included.

A total of 233 genotypes were genotyped with 15 SSR markers, which were proposed by the ECPGR [21]. All SSR loci analysed in this study showed a high degree of polymorphism with 11 to 23 alleles per locus. Compared to other studies on genetic diversity in apples, the average number of alleles per locus (15.2) was similar to some other studies, e.g. 16.7 [21], 16.9 [50], 14.6 [22] and 14.3 [36]. Several factors could be responsible for the differences in estimates, the most likely being simply differences in sample size and loci analysed. The effective number of alleles per locus was 6.47, which is similar to the value of 6.69 for Spanish collection [21] and higher than for Norwegian (5.43; [36]) and Italian germplasm (5.94; [22]). The difference between the average number of alleles and the average number of effective alleles could be due to the presence of rare alleles. Both the observed and expected heterozygosity values were high regardless of the SSR marker, suggesting that the collection was highly diverse. Compared to other studies, the mean He value observed in our study (0.83) was similar to the values of 0.82 for Spanish collection [21], 0.83 for germplasm in Bosnia and Herzegovina [50] and 0.80 for the collection of north-western Spain, Portugal and Canary Islands [51]. It is noteworthy that the mean values of observed and expected heterozygosity for the microsatellite markers in our apple population show a remarkable similarity. Since the observed heterozygosity is very similar to the expected values, this indicates that our sample accurately reflects the genetic diversity present in the population. However, the observed heterozygosity exceeded the expected values at most loci, suggesting low inbreeding and the presence of admixed genotypes. Such consistency reinforces the reliability of our data and suggests a balanced genetic structure among the microsatellite loci, a promising sign of genetic diversity in our apple population, as reported in the studies by Lassois et al. and Urrestarazu et al. [18, 21]. In addition, the overall mean PD (discrimination power) of 0.94 (with a range of 0.86 to 0.98) suggests that the loci are polymorphic enough to discriminate individuals. According to Liang et al. [50] for Italian apple germplasm and Ferreira et al. [52] for Portuguese apple germplasm, also in our germplasm the variation of Fi values within the set of varieties, depending on the observed locus, indicates the absence of close groups of related individuals within the analysed germplasm. This is consistent with the forced allogamy caused by the self-incompatibility of *Malus x domestica*. Given all these parameters showing a high level of genetic diversity in Slovenian germplasm, it is of particular importance to include these genotypes in international germplasm databases as well, as they provide a broad reservoir of alleles for apple breeding and improvement. Such diversity increases the potential to identify genotypes with resistance to pests, diseases and abiotic stress, which is crucial for adaptation to changing climatic conditions.

Local varieties are well adapted to specific environmental conditions and agricultural practices and often show some diversity in morphological and physiological traits compared to the originals, so they have different names. The correct identification of genotypes emphasises the need to study germplasm collections with powerful tools such as molecular markers. In particular, SSR markers have been used in genetic diversity studies and clarified cases of synonymy and homonymy in core collections [18, 19, 21, 22, 36]. In brief, our study showed that 51 genotypes were duplicates and synonyms and 26 groups were homonyms. In germplasm collections, misidentification and mislabelling are considered to be the main causes of homonyms. These types of misidentification have been documented in the apple germplasm of Italy and Spain [45, 50] and are caused by changes in the names of varieties after they have been introduced into other collections and countries. Of the 51 genotypes of duplicates and synonyms, 24 genotypes were considered as duplicates, because the SSR profiles and names were identical, and 27 genotypes had to be considered as synonyms because they had the same SSR profile but different names at all 15 loci. The redundancy of 22% based on the duplicates and synonyms will reduce the cost and the labour required to maintain the revised collection. The percentage of redundant genotypes varies widely in the literature. There was 37% redundancy in the Spanish collection [21], 34% redundancy in the Italian collection [50], 34% in the French collection [18], 16% redundancy in 14 large European apple collections [24]. The synonyms in this study are based on the molecular profile and do not take into account the phenotypic variation between the synonymous genotypes, so there might be some clonal variation.

In this study, the genetic structure of apple germplasm in Slovenia was analysed using a UPGMA dendrogram and Bayesian analysis of genetic structure. The UPGMA dendrogram grouped genotypes and reference varieties into three main groups (A, B and C) reflecting hierarchical relationships based on genetic distance. Bayesian clustering resulted in two main clusters (K = 2). These clusters broadly corresponded to the geographical origin of the collections, with genotypes originating mainly from the national repository in Pleterje (G2) on one side and genotypes from the Kozjansko Regional Park (G1) on the other. Admixture was observed in several individuals, indicating a continuous gene flow between the populations. This is consistent with the historical exchange of plant material with neighbouring regions such as Italy and Austria, as well as with other countries in Central and Eastern Europe countries through the migration of seasonal workers [53]. A second level of clustering was proposed, further dividing the genotypes into four (K = 4) and two (K = 2) subgroups, respectively. Two main groups were also identified in collections of local apple varieties from Aragon [54] and north east Spain [21]. Taken together, these results shed light on the genetic diversity within the apple germplasm and provide important insights for conservation and breeding strategies.

The majority of cultivated apple varieties are diploid (2x = 34), but a few apple varieties are triploid (3x = 51) and were also found in the analysed collections. The detection of triploids using both approaches, SSR markers and flow cytometry, provided valuable insights into the genetic composition of the analysed apple germplasm. While 41 genotypes (23%) were confirmed as triploid by flow cytometry, 11 genotypes (Gloria Mundi, Bismarckapfel, Jerseyred, Zelenec 1, Damason Renette, Vrtnica, Bohnapfel, Lovrenčovka, Bartoloenka, Orehovec, Zimski Rambour), which were originally identified as triploid by SSR profiling, could not be confirmed by flow cytometry, as the samples were of insufficient quality to determine the ploidy level. In addition, these discrepancies may be due to technical artefacts in SSR genotyping, a duplication event, or a somatic mutation that produces a chimeric or mosaic state in diploids [50, 55]. Therefore, verification of ploidy level by flow cytometry contributes to more reliable results. A similar percentage of genotypes was found in studies with Spanish (24%; [21]), Bosnian (27%; [19]), Spanish and Portuguese (24%; [51]) apple germplasm. Triploid apple varieties are particularly valued by growers for their larger flowers, fruit size, and increased biomass, are more vigorous and have better resistance to abiotic and biotic stresses [56, 57], which explains the higher incidence of triploids in apple collections influenced by grower preferences. Lassois et al. [18] also found that triploids are more common in older varieties than in more recently developed varieties. This can be confirmed by the fact that the triploid varieties obtained in older breeding programs were the only and valuable way to increase yield with the existing cultivation technology. The continued presence of triploid apple varieties in these older varieties emphasises their importance. Their preservation through clonal propagation therefore ensures the preservation of valuable traits. In addition, studying adaptability of these old local genotypes provides information on the resilience of the germplasm under changing environmental conditions. Moreover, these characteristics of triploid plants are attractive for breeders to breed triploid plants that are more resistant to stress and have a higher nutritional value.

## Conclusion

In this study, microsatellite markers were used for the first time to genotype a collection of apple genotypes in Slovenia, providing valuable insights into their genetic diversity, structure and relationships. Knowledge of genetic diversity among genotypes is crucial for the effective utilisation and conservation of traditional and local material. The use of SSR markers helped to identify duplicates, synonyms and homonyms within and between collections, information that is highly valuable for the cost-effective management of germplasm. The analysed genotypes showed a high level of genetic diversity and the identification of genetically unique local genotypes provides a strong foundation for establishing a representative core collection. Such a collection will support breeding programmes aimed at improving adaptability, fruit quality and disease resistance under future climate conditions.

## Data Availability

The data that support the findings of this study are not openly available due to reasons of sensitivity and are available from the corresponding author upon reasonable request.
